# Real-life use of ropeg-interferon α2b in polycythemia vera: patient selection and clinical outcomes

**DOI:** 10.1007/s00277-024-05809-6

**Published:** 2024-05-21

**Authors:** Francesca Palandri, F. Branzanti, M. Venturi, A. Dedola, G. Fontana, M. Loffredo, A. Patuelli, E. Ottaviani, M. Bersani, M. Reta, O. Addimanda, V. Vicennati, N. Vianelli, M. Cavo

**Affiliations:** 1 IRCCS Azienda Ospedaliero-Universitaria di Bologna, Istituto di Ematologia “Seràgnoli”, Bologna, Italy; 2 Department of Medical and Surgical Sciences (DIMEC), Alma Mater Studiorum University of Bologna, Bologna, 40138 Italy; 3 UO Interaziendale Medicina Interna ad Indirizzo Reumatologico AUSL BO-IRCCS AOUBO, Bologna, Italy; 4 Division of Endocrinology and Diabetes Prevention and Care, IRCCS Azienda Ospedaliero-Universitaria di Bologna, Bologna, Italy

**Keywords:** Polycythemia vera, Ropeg-interferon α2b, Cytoreduction, Real-life, Myeloproliferative neoplasms

## Abstract

Ropeginterferon-alfa2b (ropegIFNα2b) is a long-acting IFN formulation with broad FDA/EMA approval as a therapy of polycythemia vera (PV) with no symptomatic splenomegaly. There is currently lack of information on the real-world patient selection, including the impact of local reimbursement policies, and drug management, particularly: type/timing of screening and follow-up tests; absolute/relative contraindications to therapy; ropegIFNα2b dose and combinations with hydroxyurea. As a sub-analysis of the PV-ARC retrospective study (NCT06134102), we here report our monocenter experience with ropegIFNα2b in the period from January 2021, corresponding to drug availability outside clinical trial, and December 2023. Among the 149 patients with EMA/FDA indication, only 55 (36.9%) met the local reimbursement criteria and 18 (12.1%) received ropegIFNα2b. Thanks to appropriate screening, relative/absolute contraindications to ropegIFNα2b were detected and managed in a multidisciplinary manner. Efficacy and safety of ropegIFNα2b was confirmed, with 3 cases of early molecular response. General use of low ropegIFNα2b dose, with frequent need for hydroxyurea combinations, was noted. This real-world experience suggests a significant impact of local regulations on drug prescription and the need for greater real-world data collection on ropegIFNα2b in PV patients. Also, it describes appropriate multidisciplinary screening and monitoring procedures during ropegIFNα2b therapy.

**Supplementary Information:**

The online version contains supplementary material available at 10.1007/s00277-024-05809-6.

## Introduction

Polycythemia Vera (PV) is a Philadelphia-negative chronic myeloproliferative neoplasm (MPN) characterized by erythrocytosis and increased risk of thrombosis and evolution into myelofibrosis (PPV-MF) or acute myeloid leukemia (AML). Thrombocytosis, leukocytosis, splenomegaly, and systemic symptoms may also occur [1].

PV therapy includes low-dose aspirin and phlebotomies (target hematocrit < 45%) in all patients. In high-risk patients, (i.e., age > 60 and/or history of thrombosis), the addition of cytoreductive therapy is indicated. Recently, the European Leukemia Net (ELN) recommended cytoreduction also in patients at low thrombotic risk carrying additional criteria for therapy start (persistent or progressive thrombocytosis, leukocytosis, splenomegaly, symptoms; uncontrolled hematocrit and/or phlebotomies intolerance) [2].

First-line cytoreductive therapy includes hydroxyurea (HU) and interferons (IFN) [3]. Ropeginterferon-alfa2b (ropegIFNα2b) is a pegylated recombinant human IFN with a subcutaneous administration every two weeks. It is approved for the treatment of PV with no symptomatic splenomegaly, based on the results of the PROUD-PV/CONTINUATION-PV studies, that included a population of “early stage” PV, naïve for cytoreductive therapy or under HU for less than three years and not in complete response. Patients were randomized to receive either ropegIFNα2b or HU [4]. The complete hematologic response (CHR) was achieved faster in patients treated with HU. However, from the 2-year timepoint onwards, rates were significantly higher in the ropegIFNα2b arm, with consistent reduction of phlebotomies need. Molecular responses were also superior, with around 20% of ropegIFNα2b patients having a *JAK2*^V617F^ variant allele frequency < 1% at 6 years [5]. RopegIFNα2b shared common interferon-related toxicities (autoimmune diseases, mood depression) but with overall good safety profile and no excess toxicity compared to HU [4]. More importantly, the rate of thrombosis was comparable across the two treatment arms. In the “low-PV” clinical trial, a better hematocrit control compared to phlebotomies alone was showed in low-risk patients receiving ropegIFNα2b [6].

Over the last decades, efficacy and safety of many IFN formulations in PV have been variously reported, mainly based on clinical trials or off-label use [7, 8]. However, there is dearth of real-world data on the role of ropegIFNα2b after its approval in PV. Particularly, information is scant on: (1) type of screening examinations and impact of baseline autoimmune diseases or laboratory abnormalities on decision to treatment start and its safety; (2) modalities of transition from another IFN formulation to ropegIFNα2b, and results after switching; (3) ropegIFNα2b dose and combination with HU; (4) use in low-risk PV.

With these aims, we here report our clinical real-life experience on the use of ropegIFNα2b in PV patients.

## Methods

Clinical and laboratory data were collected as a subgroup monocenter analysis of the PV-ARC study (NCT06134102) [4]. PV diagnosis was made according to 2022 WHO criteria [10]. Treatments and clinical/laboratory tests were performed according to standard practice, at discretion of the treating hematologist.

CHR was defined according to ELN criteria: hematocrit (Hct) < 45% without phlebotomies (PHL) for 3 months; platelets ≤ 400 × 10^9^/L; white blood cells (WBC) count ≤ 10 × 10^9^/L, and normal spleen size [11]. JAK2^V617F^ variant allele frequency (VAF) was assessed in granulocyte DNA by quantitative PCR-based allelic discrimination assay (ipsogen JAK2 MutaQuant Kit, QIAGEN, Marseille, France) on 7900 HT Fast Real Time PCR System (Applied Biosystem, Life Technologies, Carlsbad, CA, USA) [12]. The other variants were searched by ultra-deep Next Generation Sequencing (NGS) using the commercial Myeloid Solution by Sophia Genetics, a panel designed for the identification of mutations in 30 genes associated with Myelod Neoplasms [13]. Molecular response was defined as > 50% VAF reduction compared to baseline. Mood disorders were evaluated by the Hospital Anxiety and Depression Scale (HADS), that includes 14 questions that measures anxiety and depression (7 questions each, scored zero to three), with a maximum score of 21 for anxiety or depression [14]. Drug tolerability was graded according to CTCAE v5.0. Non melanoma skin cancers (NMSC) were defined and diagnosed according to standard criteria [15].

Statistical analysis was carried out at the biostatistics laboratory of the MPN Unit at the Institute of Hematology “L. and A. Seràgnoli”, IRCCS Azienda Ospedaliero-Universitaria di Bologna. Continuous variables have been summarized by their median and range, and categorical variables by count and relative frequency (%) of each category.

## Results

### Patient cohort

In a total cohort of 198 patients who are currently in clinical follow-up at our MPN Unit, 175 have a potential indication for cytoreductive therapy (144 high-risk and 31 low-risk patients). Specifically, criteria for therapy start in these low-risk patients were: persistent/progressive leukocytosis (100% increase if WBC < 10 × 10^9^/L or 50% increase if WBC > 10 × 10^9^/L or WBC > 15 × 10^9^/L at diagnosis and HU start), n. 8 (25.8%); extreme persistent thrombocytosis (PLT > 1000 × 10^9^/L at diagnosis and HU start), n. 1 (3.2%); progressive splenomegaly (increase of > 5 cm from diagnosis), n. 4 (12.9%); inadequate Hct control (> 6 phlebotomies/year; Hct > 53% at diagnosis and HU start; PHL intolerance), n. 18 (58.1%) .

Overall, 152 patients (86.9%) were receiving cytoreductive therapy at last contact. According to the indications of the European Medical Agency (EMA), 149 out of these 152 patients (98.0%) could be eligible for ropegIFNα2b. Indeed, only three patients had a symptomatic splenomegaly.

Notably, only 55 patients (36.9%) met the Italian criteria for drug reimbursement, which include females with motherhood desire (n. 1, 1.8%), individuals with previous episodes of NMSC (n. 15, 27.3%) and HU-intolerant patients (n. 39, 70.9%) [16].

From the time of real-life availability in Italy of ropegIFNα2b (January 2021) to December 2023, 37 patients (24.3%) were evaluated for initiation of ropegIFNα2b; 6 out of 94 (6.4%) patients who lacked reimbursement criteria and 31/55 (56.4%) in whom ropegIFNα2b was reimbursable.

Overall, 18 patients started such therapy and 4 patients are starting ropegIFNα2b soon.

The reasons for not starting ropegIFNα2b in the 15 screened patients were: non-reimbursement of the drug (no. 5); patient refusal (no. 4; these patients did not receive any cytoreductive agent); clinical contraindications that became apparent during the screening phase (no. 6). These contraindications included anxious-depressive syndrome (HADS score ≥ 11, followed by psychiatric evaluation which discouraged use of interferons) in 4 patients (26.7%), autoimmune glomerulonephritis (collegially evaluated with treating nephrologists) in one patient (6.7%) and a thrombotic anti-phospholipid syndrome (APS), complicated by venous cerebral thrombosis and splanchnic vein thrombosis that occurred before the diagnosis of PV, in one patient. This patient, who did not meet reimbursement criteria, received collegial evaluation with treating rheumatologists and was finally treated with hydroxyurea as cytoreductive agent.

Overall, 24 patients with potential ropegIFNα2b reimbursement were not evaluated for ropegIFNα2b. Specifically, 6 patients were HU-intolerant but were allocated to other therapies (busulfan, 2 elderly patients; ruxolitinib, 4 patients with high symptoms burden); 4 HU-intolerant patients refused to start other cytoreductive therapies; 14 (58.3%) patients in good response to HU, who developed one single NMSC.

**Fig. 1:**
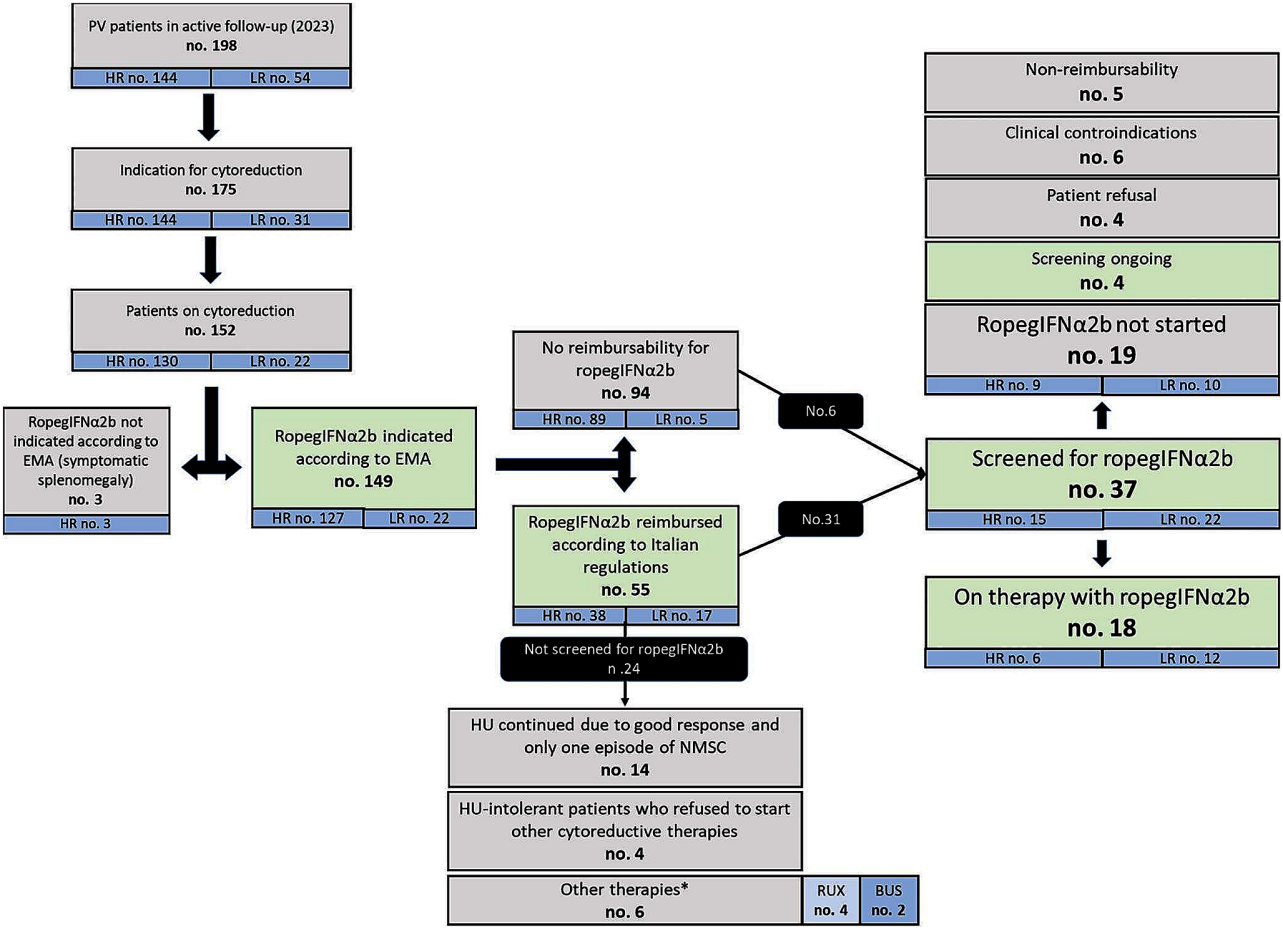
Patients’ disposition. RUX: ruxolitinib; BUS: busulfan; HU: hydroxyurea; NMSC: non-melanoma skin cancer

Patient disposition is shown in Fig. 1.

Table 1 reports the characteristics of the 18 patients who received ropegIFNα2b between January 2021 and December 2023. A graphical representation of each individual patient history has been reported in Supplemental Fig. 1.

**Table 1:** Patients characteristics at ropegIFNα2b start and outcome during therapy. Patients have been subgrouped according to thrombotic risk at ropegIFN start (low risk: age < 60 years and no previous thrombosis; high-risk: age > 60 years and/or previous thrombosis)

Characteristics at ropeg-IFN start	Low risk (*n*. 12)	High risk (*n*.6)
Male sex, n. (%)	8 (66.7%)	5 (83.3%)
Age, *years*, median (range)	47.0 (38.5–58.6)	64.9 (52.8–79.3)
JAK2 V617F VAF, *%*, mean (range)	57 (14–89)	17 (3–80)
Hemoglobin, g/dl, median (range)	14.3 (11.9–16)	12.9 (12.1–16)
Phlebotomies per year, median (range)	3 (1–16)	4.5 (1–12)
Leukocyte count, x*10*^*9*^*/L*, median (range)	7.5 (5.5–22.5)	7.5 (5.4–17.8)
Platelet count, x*10*^*9*^*/L*, median (range)	495.5 (227–917)	485.2 (192–832)
Previous thrombosis, n. (%)	0	2 (33.3%)
Cardiovascular risk factors (CVRF), n. (%)	5 (41.7%)	5 (83.3%)
Previous therapy with HU, n. (%)	8 (66.7%)	6 (100%)
Previous therapy with alternative IFN formulations, n. (%)	5 (41.7%)	0
Complete Hematological Response (CHR)	9 (75.0%)	3 (50.0%)
Time to CHR, *months*, mean (range)	9.33 (1–27)	7.67 (2–11)
Maximum ropeg dose, *mcg/2 weeks*, mean (range)	131.3 (50–200)	120.8 (100–200)
Dose increase, n (%)	9 (75.0%)	2 (33.3%)
Thrombosis during ropegIFN, n. of patients (%)	0	1 (16.7%)
Follow-up from ropegIFN, *months*, mean (range)	24.8 (3.3–34.8)	18.1 (4.4–36.1)

### Screening examinations and impact of baseline laboratory abnormalities on ropegIFNα2b safety

In all patients, a complete baseline screening, including patient history and comorbidities, biochemistry, autoimmune serology, thyroid function, mental health assessment was performed. These evaluations were repeated during therapy at regular timepoints. Baseline and follow-up evaluations are detailed in Table 2.

**Table 2:** Clinical screening and follow-up tests during ropegIFNα2b therapy

Patient evaluation	Timing	Further evaluation	Multidisciplinary referral
Complete Blood count	Every month until Hct < 45%, then every 3 months	Differential leukocytes count to exclude leuko-erythroblastosis	
Liver function tests	Every 3 months in the first 6 months, then every 6–12 months	Exclude viral or autoimmune hepatitis	Hepatologist
Renal function tests	Every 6 months		Nephrologist
Thyroid function – *Thyroid-stimulating hormone (TSH)* – *Anti-thyroglobulin antibodies (anti-TG)* – *Anti-thyroperoxidase antibodies (anti-TPO)*	Every 3 months in the first 6 months, then every 6–12 months	Thyroid echography	Endocrinologist
General autoimmunity screening – *Antinuclear Antibodies (ANA)* – *Rheumatoid factor*	Every 12 months or in case of symptoms suggestive for autoimmune disease	Extractable Nuclear Antigen Antibodies (ENA) Anti-mitochondrial antibodies (AMA) Anti-double stranded DNA antibodies (Anti-dsDNA)	Rheumatologist
Anti-phospholipid antibodies – *Lupus anticoagulant* – *Anticardiolipin antibodies* – *Anti-ββ glycoprotein I (aββGPI) antibodies*	Every 12 months or in case of symptoms suggestive for active disease	Exclude anti-phospholipid syndrome	Rheumatologist
Mental Health – *Patient history including previous mood disorders* – *Hospital Anxiety and Depression Scale (HADS)*	Every 6–12 months or in case of symptoms suggestive for mood disease		Psychiatrist
Eye health – *Known retinopathy or diseases that may be associated with retinopathy (i.e., diabetes, hypertension)*	According to standard follow-up		Ophthalmologist
Heart function – *Medical history investigating recent cardiac disease*	According to standard follow-up	ECG, Echocardiography	Cardiologist

At the start of ropegIFNα2b, 6 (33.3%) out of 18 patients who received ropegIFNα2b had autoimmune diseases or laboratory abnormalities that were not considered sufficient to contraindicate the initiation of therapy (anti-thyroglobulin auto-antibodies with normal TSH, 3 cases; anti-nuclear antibodies, titer 1:160, with no rheumatological symptoms, 2 cases; seronegative oligoarthritic disease not requiring active therapy, 1 case).

Notably, 2 out of 3 patients with anti-thyroglobulin auto-antibodies developed a clinically significant thyroiditis requiring levothyroxine therapy after 1.1 and 2.8 years of ropegIFNα2b therapy, respectively. All other patients had no occurrence or progression of autoimmune disease over time (mean time on ropegIFNα2b: 15.8 months). Other adverse events during ropegIFNα2b were flu-like syndrome (2 cases); alopecia and xerophthalmia (2 cases); creatinine increase, skin rash, migraine (1 case each). These events were all grade 1–2, transient and manageable with temporary dose reductions. All patients continued ropegIFNα2b at last contact.

No case of second solid tumors, NMSC or disease evolution to PPV-MF or AML have occurred.

### Switch from other interferon formulations to ropegIFNα2b

Globally, 5 patients had received an alternative IFN formulation before starting ropegIFNα2b (specifically, pegIFNα2a: 2 patients; IFNα2b: 3 patients). In 4 patients, IFN was discontinued due to intolerance, particularly two cases of flu-like syndrome, one case of transaminase increase, and one case of persistent migraine. None of these toxicities occurred after ropegIFNα2b switch. PegIFNα2a was discontinued in one patient due to resistance (persistent massive thrombocytosis and pruritus), which both improved after ropegIFNα2b start.

In two patients, there was a time interval between the discontinuation of the other IFN formulation and the start of ropegIFN. In 3 patients, ropegIFNα2b was sequential to another non-pegylated interferon. In absence of standardized recommendations, starting dose of ropegIFNα2b was empirically set at 100mcg/2 weeks in all patients.

### RopegIFNα2b dose and combination therapy with hydroxyurea

In 7 patients, HU was used in combination with ropegIFNα2b for a mean time of 5 months (range: 0.25–11). In 6 cases, HU was used in combination from the start of ropegIFNα2b therapy; in one case, HU was introduced after 8 months of ropegIFNα2b therapy, due to persisting thrombocytosis. The combination was necessary due to the persistence of at least one of the following conditions: extreme thrombocytosis (4 cases), uncontrolled hematocrit (1 case), leukocytosis (1 case) and severe itching (2 cases). The combination was ongoing in 4 patients at last contact due to insufficient hematological response.

Overall, 11 patients required a ropegIFNα2b dose increase, which was performed after a median time of 9.4 months (range 2–28). Reasons for dose increase included: persistent thrombocytosis (6 cases), uncontrolled hematocrit (4), persistent pruritus (3). Maximum ropegIFNα2b dose was 200 mcg/2 weeks.

### Response to ropegIFNα2b therapy

In all patients, a progressive decrease in leukocytes, platelet count, phlebotomies need, and symptoms were observed over time (Supplemental Fig. 2).

None of the patients included in the analysis were in CHR prior to the start of treatment with ropegIFNα2b.

Overall, 12 (66.7%) patients achieved a CHR, after a mean time of 8.9 months (range 1–27). Notably, in two cases, the CHR was achieved during combination therapy with HU, in patients who started ropegIFNα2b after 2 and 13 months of HU; since CHR was never achieved during HU monotherapy, the hematological response was mainly attributed to ropegIFNα2b.

All patients maintained the CHR at last follow-up. Six (33.3%) patients had not achieved a CHR after a mean time on treatment of 17.2 months (range 7–32). Mean ropegIFNα2b dose was 122.9 and 137.5 microg/2 weeks in responsive and non-responsive patients, respectively.

Median TSS decreased from 6.5 (range 0–36) at baseline to 0 (range 0–37) at 6 months. In two out of three patients with baseline itching, this symptom improved during ropegIFNα2b.

A molecular response was observed in 3 patients in sustained CHR. Specifically, JAK2^V617F^ VAF decreased from 69.5%, 89%, and 16–30%, 4%, and 8%, respectively, after 11.7, 18.4 and 17.7 months of therapy. In the remaining cases, JAK2^V617F^ VAF had only mild fluctuations (± 10% from baseline).

NGS was performed in 7 patients before and after 12–24 months of ropegIFNα2b. No sub-clonal mutations were detected at baseline or emerged during therapy.

### Use of ropegIFNα2b according to risk category

Altogether, 12 patients (66.6%) started ropegIFNα2b while at low thrombotic risk. Triggers for therapy start were: extreme thrombocytosis (> 1000 × 10^9^/L in at least two evaluations) (6 cases), excessive number of and/or intolerance to phlebotomies (8), persistent/progressive pruritus (3), hyperleukocytosis (> 15 × 10^9^/L in at least two evaluations) (2). In 7 patients, HU was used before ropegIFNα2b and then discontinued due to intolerance (4 patients) and/or resistance (3 patients).

On the other hand, high-risk patients started ropegIFNα2b because of intolerance (5 cases) and resistance (1 case) to HU. In these patients ropegIFNα2b was preferred over ruxolitinib because of concomitant NMSC (1 case), young age (< 60 years) (1 case) and patient preference (4 cases).

The percentages of CHR were higher in low-risk patients (75% versus 50% in high-risk patients). Low-risk patients also received higher ropegIFNα2b doses (max dose 131.3 vs. 120.8 µg/2 weeks) and received more frequently a dose increase (75.0% vs. 33.3%).

Safety was comparable.

## Discussion

This real-life study highlights, for the first time, that while the EMA indication for ropegIFNα2b includes almost all patients with PV (with the exception of subjects with symptomatic splenomegaly, who represent about 1–3% of the total population requiring cytoreduction), the reimbursement granted by our local regulatory authorities is much more restrictive (women desiring pregnancy, patients with previous skin cancer or with intolerance to HU) and includes, in our original research, only 26.6% of patients in need of cytoreduction. Also, reimbursement issues motivated the non-use of ropegIFNα2b in many patients who did not receive the drug after initial evaluation. This finding highlights the need for greater data collection and possible re-evaluation of local reimbursement restrictions, that are so far mainly motivated by scarce real-world knowledge of ropegIFNα2b and conversely by the availability of robust efficacy and safety data of HU and ruxolitinib [17].

Notably, all patients who received ropegIFNα2b after a previous IFN formulations reported better tolerance, absence of cross-toxicity and, in one patient, also better clinical efficacy after switching. This is possibly due to prolonged half-life of ropegIFNα2b, but also to the use of relatively low doses, with delayed dose escalation and need for HU combination in many patients. Higher doses of ropegIFNα2b were administered to low-risk patients, who were considered suitable for a more aggressive therapeutic approach due to younger age and lower burden of comorbidities. This has resulted in a higher rate of CHR, confirming a correlation between dose and response.

While our experience demonstrates that such combination is feasible and safe, the optimization of ropegIFNα2b use with avoidance of drug underdosing both at therapy start and in case of suboptimal response, could maximize the therapeutic benefit [18]. Indeed, higher doses of ropegIFNα2b recently showed a 61.2% of CHR rate at week 24, with acceptable but not negligible toxicity [19].

There is no international consensus on how to screen a patient before ropegIFNα2b therapy, particularly in terms of autoimmune disease and mood disorders, how to manage eventual abnormalities, or the timing of periodic re-evaluations. It is generally recommended to exclude autoimmune thyroiditis and subclinical autoimmune diseases, with referral to the endocrinologist/rheumatologist when necessary [18]. In our experience, we have also included the use of the HADS scale for early recognition of mood disorders, and in 4 cases ropegIFNα2b was excluded based on high HADS score and psychiatric counselling. We have also implemented the screening for anti-phospholipid syndrome (APS), a pro-thrombotic autoimmune syndrome that is highly linked to IFN release, and that therefore may be exacerbated by interferon-based therapies [20, 21]. A prospective evaluation of these autoantibodies may provide more reliable data on the incidence of these alterations in PV patients and also on the possible impact of ropegIFNα2b on this immune-mediated disorder [22].

Our cohort is too small to provide insights on hematological and molecular response to ropegIFNα2b. However, despite short follow-up, it can be emphasized that the CHR rates obtained in our analysis are quite comparable to those reported in the PROUD-PV trial (66.7% vs. 55.6%) [4]. Also, in low-risk patients we observed a CHR of 75%, comparable to the 84% reported in the LOW-PV trial [6].

In addition, we noted 3 patients achieving a molecular response, with 2 patients with VAF < 10% after less than 2 years from therapy start. It was previously shown the presence of additional sub-clonal mutations may be associated with lower pegylated IFNα efficacy in CALR-mutated ET [23]. Also, patients with ET/PV carrying both JAK2 and TET2 mutations at IFN start had less significant reduction in JAK2V617F allele burden compared with JAK2 mutant/TET2 wild-type patients [24]. Finally, IFN therapy was found to be ineffective in reducing the allele burden of the TET2 mutation in individual colonies grown from erythroid progenitors [25]. In our cohort, no patient had additional subclonal mutations by NGS analysis. However, the collection of data on VAF and non-driver mutations during ropegIFNα2b may be relevant for a better evaluation of patients’ prognosis and possibility of treatment discontinuation.

NMSC are the most common malignancies diagnosed in Caucasian populations [26]. In MPN patients, their incidence is significantly increased by prolonged HU and by ruxolitinib therapy [27, 28]. Recently, the ELN panel recommended the switch to a second-line therapy, preferably interferon, in PV patients who develop NMSC during HU therapy [2]. The application of this recommendation in real-world is unknown. In our cohort, only one out of 15 HU-treated patients with NMSC switched to ropegIFNα2b, mainly due to an overall good response to HU and the absence of multiple events. Whether this clinical attitude will change with more expert management of PV is to be evaluated in the future.

HU is a drug in pregnancy category D (expected to cause an increased incidence of human fetal malformations or irreversible damage) and is also linked to altered spermatogenesis [29, 30]. Its avoidance is recommended in both female and male patients with motherhood fatherhood desire; however, only females who wish to become pregnant satisfy our local regulatory criteria for ropegIFNα2b reimbursement. This gender imbalance may have severe impact on male patients’ quality of life; in our cohort, 3 out of 5 patients that were excluded from ropegIFNα2b due to lack of reimbursement criteria were young males with fatherhood desire.

Also, the ELN suggest ropegIFNα2b as front-line therapy of low-risk PV. Here we observed that this drug was mainly used in these patients, that are projected to long-term treatment and could possibly benefit more from ropegIFNα2b [31, 32].

Overall, this original research, while confirming the efficacy and safety of ropegIFNα2b, also highlights the discrepancies between patient needs and local regulations and emphasizes the need for real-world data collection to improve patient selection. Furthermore, our experience calls for a comprehensive effort to standardize the management of this drug by providing appropriate guidance for screening tests to be performed at baseline and the timing/methods of their repetition in follow-up. Finally, it will be valuable to share a standard for the management of any clinical-laboratory abnormalities that may emerge at screening or during therapy, particularly by pointing out absolute contraindications to the start or continuation of ropegIFNα2b and by encouraging multidisciplinary approaches.

### Electronic supplementary material

Below is the link to the electronic supplementary material.

Supplementary Material 1

Supplementary Material 2

## Data Availability

The data that support the findings of this study are available from the corresponding author on reasonable request.
