# Supplementary material for: Real-life use of ropeg-interferon α2b in polycythemia vera: patient selection and clinical outcomes
Source: Ann Hematol. 2024 May 21;103(7):2347–54. doi: 10.1007/s00277-024-05809-6 (PMC11224071; doi:10.1007/s00277-024-05809-6)
Supplement: Supplementary file 1 — Supplementary Material 1 [file 277_2024_5809_MOESM1_ESM.docx]

Real-life use of ropeg-interferon α2b in Polycythemia Vera: patient selection and clinical outcomes

Annals of Hematology

F. Palandri^1^, F. Branzanti^2^, M. Venturi^1,2^, A. Dedola^1,2^, G. Fontana^1,2^, M. Loffredo^1,2^, A. Patuelli^1,2^, E. Ottaviani^1^, M. Bersani^1^, M. Reta^3^, O. Addimanda^3^, V. Vicennati^2,4^, N. Vianelli^1^, M. Cavo^1,2^

1) IRCCS Azienda Ospedaliero-Universitaria di Bologna, Istituto di Ematologia “Seràgnoli”, Bologna, Italy

2) Department of Medical and Surgical Sciences (DIMEC), Alma Mater Studiorum University of Bologna, 40138 Bologna, Italy.

3) UO Interaziendale Medicina Interna ad Indirizzo Reumatologico AUSL BO-IRCCS AOUBO, Bologna, Italy

4) Division of Endocrinology and Diabetes Prevention and Care, IRCCS Azienda Ospedaliero-Universitaria di Bologna, Italy

**Corresponding Author**

Francesca Palandri

IRCCS Azienda Ospedaliero-Universitaria di Bologna

Istituto di Ematologia “Seràgnoli”, Bologna, Italy

Tel +39 051 214 3044

Fax +39 051 636 4037

e-mail: francesca.palandri@unibo.it

## Supplemental Figure 2: Trends of WBC count (a), PLT count (b), phlebotomies need (c) and symptoms (d) over the first year of ropegIFN therapy


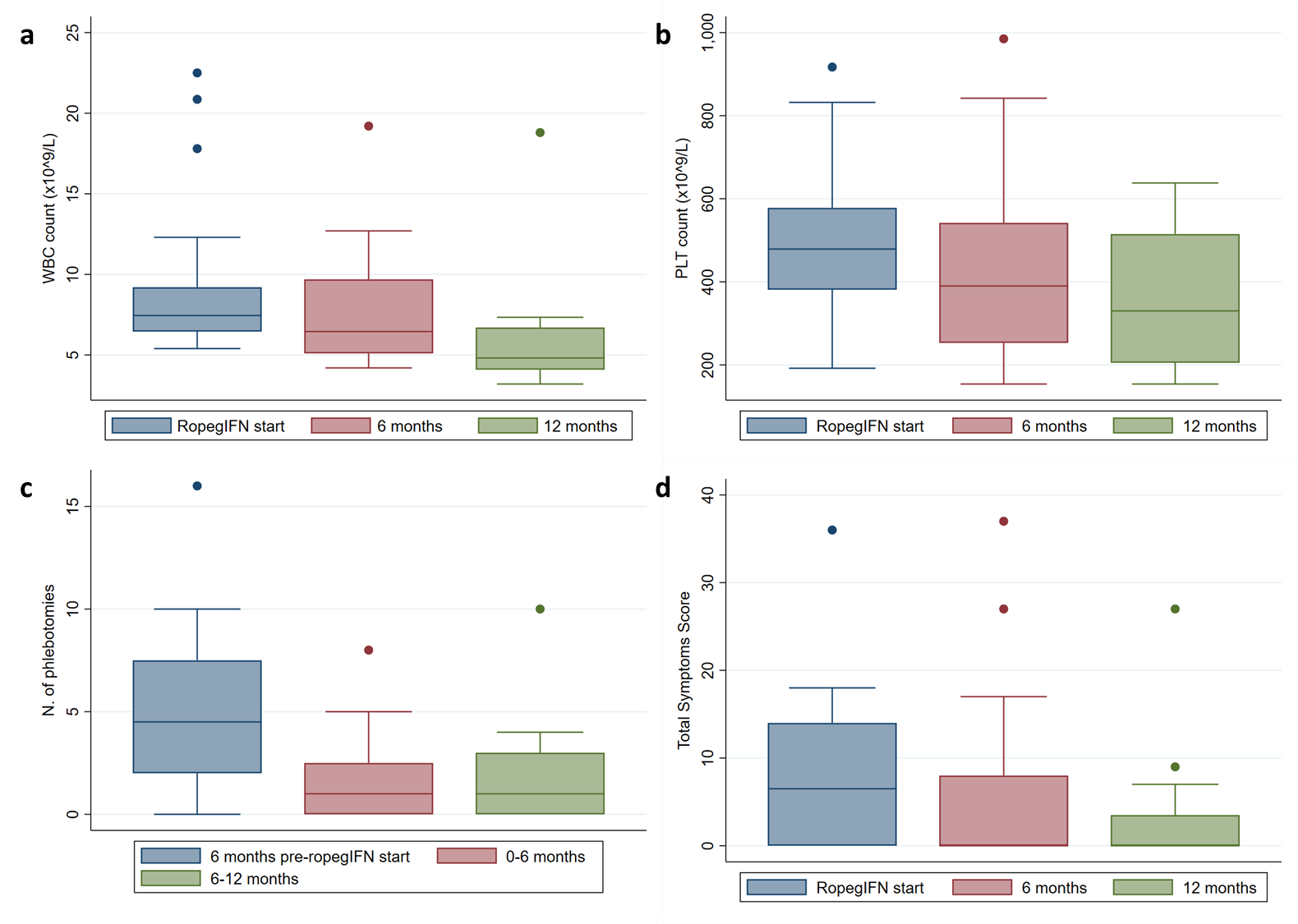


WBC: white blood cells; PLT: platelets; PHL: phlebotomies; TSS: Total Symptoms Score.
