# Supplementary material for: Real-life use of ropeg-interferon α2b in polycythemia vera: patient selection and clinical outcomes
Source: Ann Hematol. 2024 May 21;103(7):2347–54. doi: 10.1007/s00277-024-05809-6 (PMC11224071; doi:10.1007/s00277-024-05809-6)
Supplement: Supplementary file 2 — Supplementary Material 2 [file 277_2024_5809_MOESM2_ESM.docx]

Real-life use of ropeg-interferon α2b in Polycythemia Vera: patient selection and clinical outcomes

## Annals of Hematology

F. Palandri^1^, F. Branzanti^2^, M. Venturi^1,2^, A. Dedola^1,2^, G. Fontana^1,2^, M. Loffredo^1,2^, A. Patuelli^1,2^, E. Ottaviani^1^, M. Bersani^1^, M. Reta^3^, O. Addimanda^3^, V. Vicennati^2,4^, N. Vianelli^1^, M. Cavo^1,2^

1) IRCCS Azienda Ospedaliero-Universitaria di Bologna, Istituto di Ematologia “Seràgnoli”, Bologna, Italy

2) Department of Medical and Surgical Sciences (DIMEC), Alma Mater Studiorum University of Bologna, 40138 Bologna, Italy.

3) UO Interaziendale Medicina Interna ad Indirizzo Reumatologico AUSL BO-IRCCS AOUBO, Bologna, Italy

4) Division of Endocrinology and Diabetes Prevention and Care, IRCCS Azienda Ospedaliero-Universitaria di Bologna, Italy

**Corresponding Author**

Francesca Palandri

IRCCS Azienda Ospedaliero-Universitaria di Bologna

Istituto di Ematologia “Seràgnoli”, Bologna, Italy

Tel +39 051 214 3044

Fax +39 051 636 4037

e-mail: francesca.palandri@unibo.it

# Supplemental Figure 1: Patients’ timelines

**Suppl. Fig. 1A**: Low-risk patients previously treated with an alternative IFN formulation


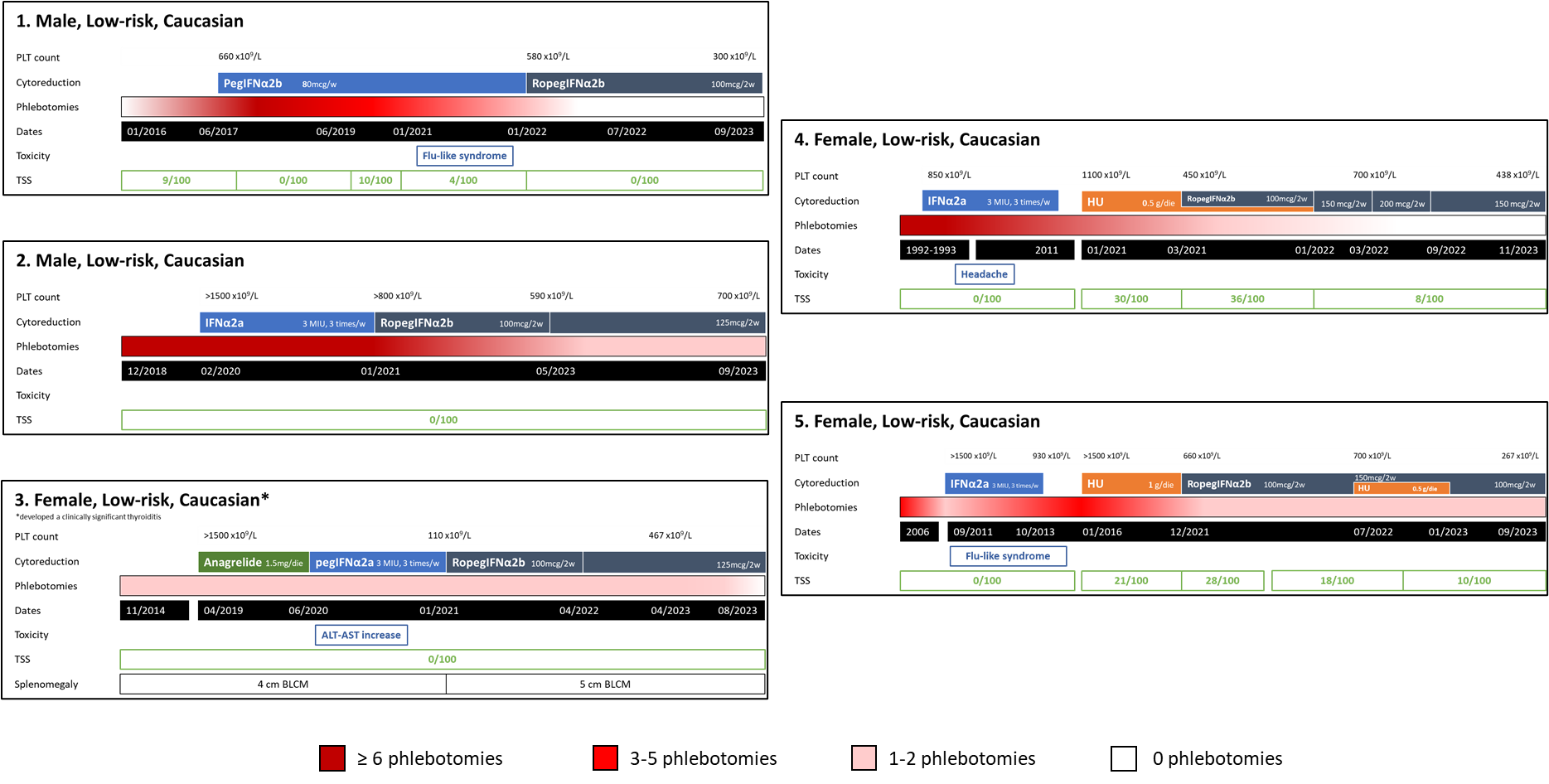


**Suppl. Fig. 1B**: Low-risk patients non-treated with an alternative IFN formulation


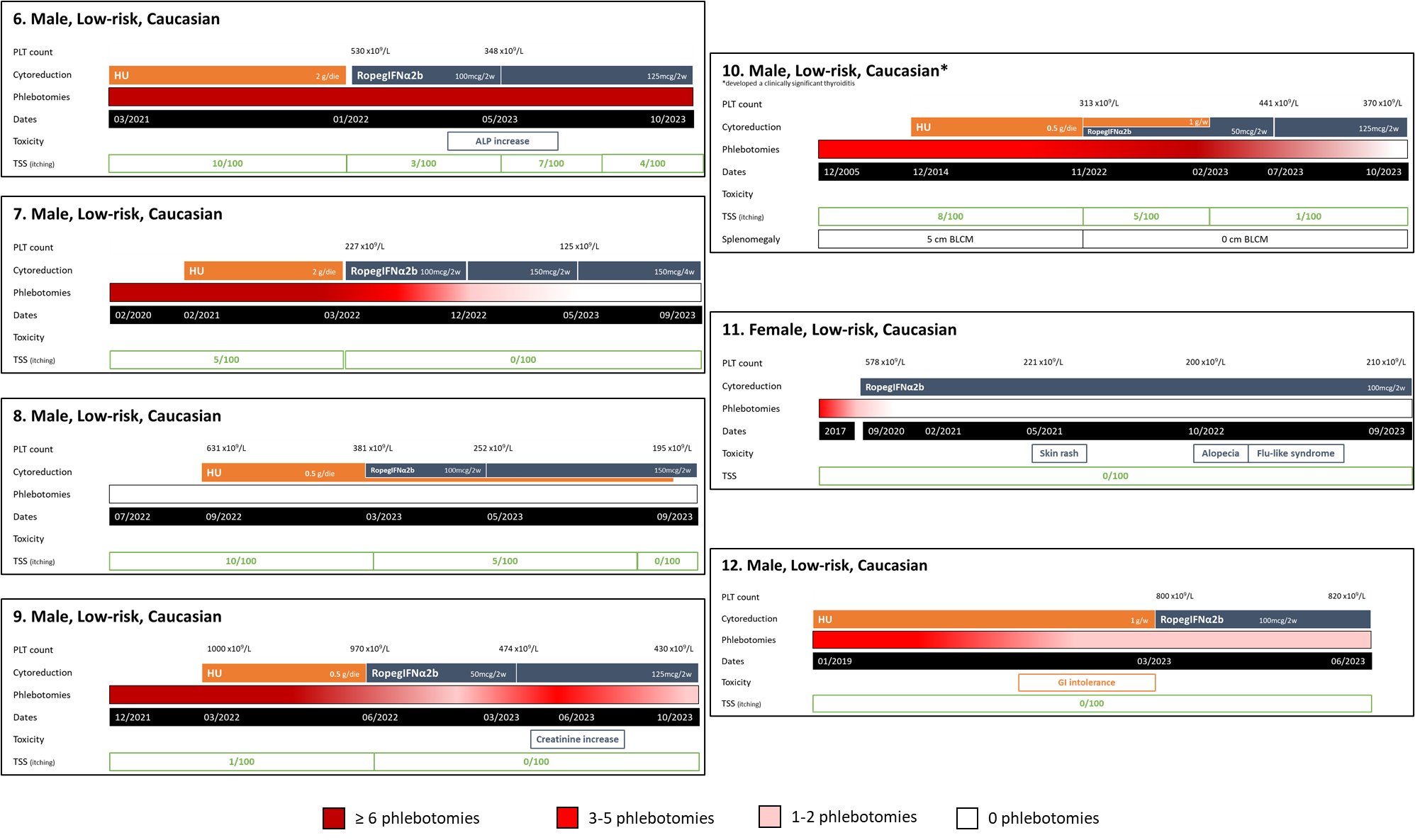


**Suppl. Fig. 1C**: High-risk patients


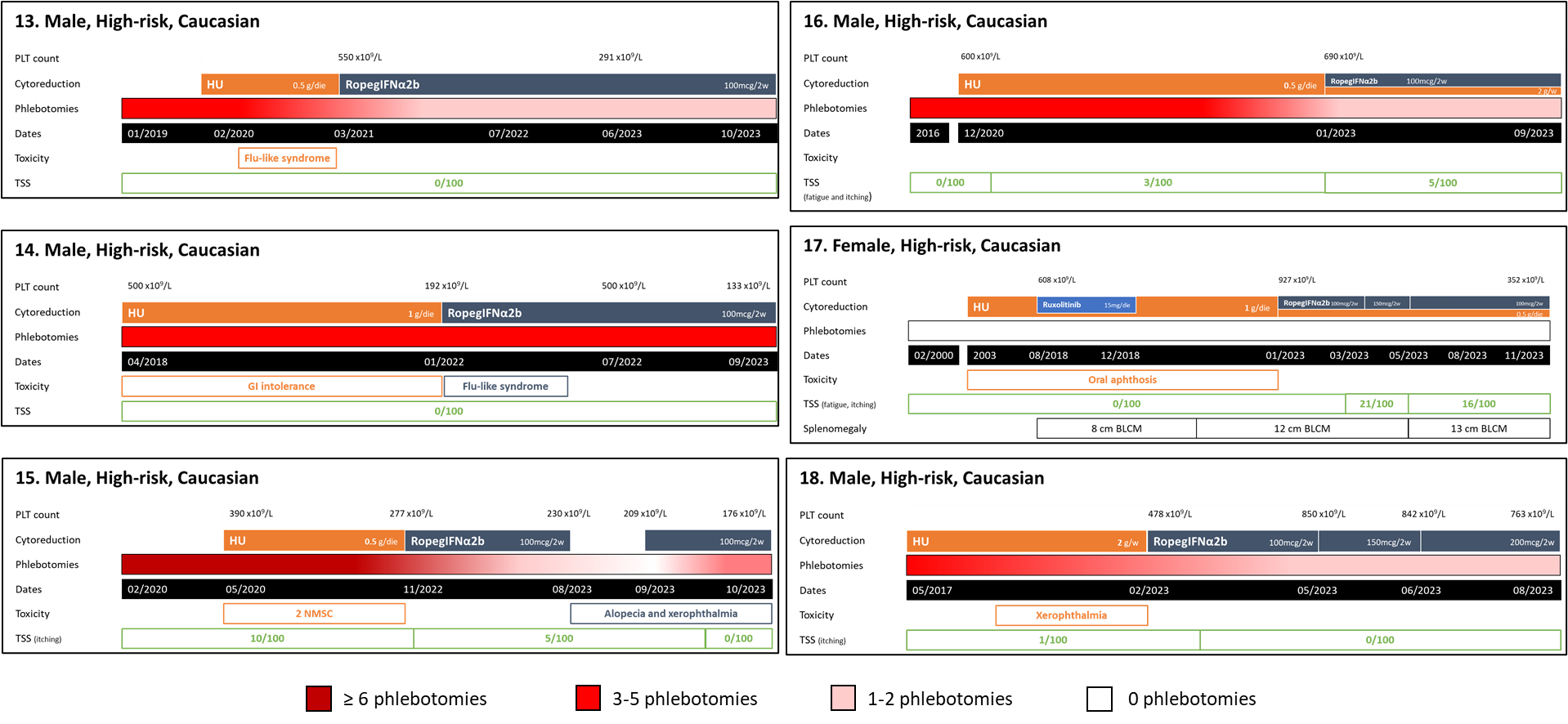


**IFN**: Interferon; **HU**: Hydroxyurea; **PLT**: Platelets; **GI**: Gastrointestinal; **TSS**: Total Symptoms Score; **PHL**: Phlebotomies; **w**: weekly; **2w**: every two weeks.

Overlapping cytoreduction bars represent joint therapies.

Patient 3 was the only one presenting splenomegaly at diagnosis.

Number of phlebotomies are reported over a 6-month period.
